# Experiences, Emotions, and Health Consequences among COVID-19 Survivors after Intensive Care Unit Hospitalization

**DOI:** 10.3390/ijerph19106263

**Published:** 2022-05-21

**Authors:** Ilenia Piras, Maria Francesca Piazza, Cristina Piccolo, Antonio Azara, Andrea Piana, Gabriele Finco, Maura Galletta

**Affiliations:** 1 Department of Biomedical Sciences, PhD School in Biomedical Sciences (Public Health), University of Sassari, 07100 Sassari, Italy; 2 Emergency Department SS. Trinità Hospital, ASL Cagliari, 09121 Cagliari, Italy; 3 Liguria Health Authority (A.Li.Sa.), 16121 Genoa, Italy; mariafrancesca.piazza@alisa.liguria.it; 4 Intensive Care Unit SS. Trinità Hospital, ASL Cagliari, 09121 Cagliari, Italy; cristinapiccolo05@gmail.com; 5 Department of Medical, Surgical and Experimental Sciences, University of Sassari, 07100 Sassari, Italy; azara@uniss.it (A.A.); piana@uniss.it (A.P.); 6 Intensive Care Unit Policlinico Universitario, Azienda Ospedaliero Universitaria Cagliari, 09042 Monserrato, Italy; gabriele.finco@unica.it; 7 Department of Medical Sciences and Public Health, University of Cagliari, 09042 Monserrato, Italy; maura.galletta@unica.it

**Keywords:** COVID-19, emotions, psychological experience, health consequences, long COVID, hospitalization, intensive care unit

## Abstract

Literature suggested that COVID-19 patients experienced hospitalization as a physically and psychologically stressful event, with the risk to develop post-traumatic stress symptoms. The study aimed to understand psychological experiences of COVID-19 survivors with severe complications during and after ICU hospitalization, and any relevant health consequences. From October 2020 to January 2021, a qualitative study was conducted in Italy via semi-structured interviews by phone or video call addressed to COVID-19 survivors, randomly enrolled among people who released their stories publicly on newspapers, television, or social media. Fifteen individuals (three women and twelve men with average age of 56.4 years) were interviewed. Four main themes emerged: (i) emotion of fear; (ii) isolation and loneliness; (iii) unawareness about the gravity of the situation as a protective factor; (iv) “Long COVID” as consequences of the disease on physical and psychological health. During hospitalization, 66.7% of participants had mild or moderate values of anxiety and depression. After discharge, 86.7% moved to normal values. The results suggest that long-COVID is an important problem to manage to improve patients’ quality of life. It is essential to guarantee a holistic take in charge starting before the discharge and continuing care after discharge in the community where they live.

## 1. Introduction

Coronavirus disease (COVID-19) is caused by the SARS-CoV-2 virus, and it determines mild or moderate respiratory illness in most infected people [1]. However, some people with demographic (e.g., elderly) and underlying medical conditions (e.g., cardiovascular disease, respiratory disease, cancer) are more likely to develop the disease in a very severe way [2,3,4]. The literature shows that the risk of being treated with a mechanical ventilator in an Intensive Care Unit (ICU) is greater in males and those with a previous history of hypertension, diabetes, and psychiatric illness, thus increasing the risk of progressing to a critical condition [4,5]. However, anyone can become seriously ill with COVID-19, with the risk of dying at any age [1] and with the possibility of survivors of severe complications having long-term effects on different organs and body systems, including pulmonary, cardiovascular, and nervous systems, as well as psychological effects [6]. According to the most internationally updated COVID-19 online case registry [7], approximately 5% of hospitalized patients with severe clinical conditions (e.g., respiratory failure) need invasive mechanical ventilation and intensive care. In the first phase of the pandemic, when little or nothing was known about the disease, being diagnosed with COVID-19 brought a feeling of disbelief, shock, and a feeling of being on one’s deathbed [8]. In fact, literature referred to the first phase of the outbreak suggested that hospitalization for COVID-19 was experienced as dramatic and physically and psychologically stressful. Most of the patients had anxiety disorders [8,9] and depression [10,11], insomnia [12,13], fear [14,15], and suffered from isolation and loneliness in the hospital due to the mandatory restriction to prevent the contagion [16]. Post-traumatic stress symptoms were also reported in clinically stable patients hospitalized for COVID-19 [12,17]. Feelings experienced by patients can likely be influenced by the severity of the clinical condition and the type of hospital setting and experience living. Patients who had severe clinical manifestations of the disease and clinical instability, required high-intensity care and were urgently transferred to ICUs from emergency departments or medical wards; they were also under pharmacological sedation and mechanical ventilation. A systematic review and meta-analysis showed that the prevalence of mortality among confirmed COVID-19 cases hospitalized in ICU was 15%, considerably higher than the overall in-hospital mortality rate (5%) of patients infected with COVID-19 [18]. Moreover, patients hospitalized in ICU are not often fully aware of what is happening (e.g., necessary invasive procedures, endotracheal intubation) due to sedation and/or altered mental status; this can reduce patients’ sense of safety, which increases the possibility of having a traumatic experience [19]. In a recent study, Sahoo et al. [20] reported that patients considered the hospitalization in ICU as a painful experience and as one of the worst phases of their life. These people were afraid of dying during hospitalization due to the uncertainty of what could happen in the ICU and after discharge due to the progression of the disease with both physical and psychosocial negative effects [21]. Literature from the last year showed that clinical sequelae can persist after the disease. This condition is currently called Long-COVID (or post-COVID syndrome) which is a complex condition with heterogeneous symptoms and complications that follow the acute phase of the infection and have a variable duration. The most common physical symptoms are dyspnea, muscle pain, joint pain, and headache [22]. Psychological symptoms are memory loss, anxiety, sleep disorders, fatigue, and cognitive impairment [23]. A recent meta-analysis including 48 studies showed that people experienced fatigue and cognitive impairment 12 or more weeks after acute illness [24]. It is understandable that these prolonged symptoms can affect one’s mood and quality of life. Although greater complexity of the patient’s psychological outcomes does not appear to be associated with the severity of the clinical condition [10] and therefore to the hospitalization setting, the prevalence of persisting symptoms is higher among survivors of COVID-19 after hospitalization in ICU [25].

Since the beginning of the pandemic, the scientific evidence related to the psychological consequences has mainly focused on objectively measurable aspects related to the clinical sphere, such as panic disorder, anxiety, depression, and post-traumatic stress disorder, neglecting the subjective experiences and emotions which may affect the psychological well-being and quality of life of patients who survived severe complications of the disease in the ICU [26,27,28].

To the best of our knowledge, in Italy there are no qualitative studies exploring the feelings and the experiences of ICU survivors with severe complications from COVID-19. Indeed, these patients experienced suddenly severe clinical complications of a disease still unknown, at the time of the study. For this reason, the present study aims to understand the individual experiences (e.g., emotions, worries, and health consequences) of survivors and to obtain relevant information about psychological experience during the ICU stay and any post-hospitalization consequences to be managed in order to encourage patient and family-centered models of care and to ensure organized, safe, and evidence-based action for future pandemics, thus minimizing negative feelings and contributing to the humanization of care.

## 2. Materials and Methods

### 2.1. Study Design

From October 2020 to January 2021, a qualitative study was conducted in Italy through the administration of a semi-structured interview to ICU survivors after severe complications due to SARS-CoV-2 virus infection (people discharged after ICU hospitalization). The research was carried out using Colaizzi’s phenomenological method [29], which focuses on participants’ experiences and feelings by finding shared patterns rather than single aspects in individual participants. This approach ensured the authenticity of the experience told by the participants for adhering to scientific standards.

### 2.2. Procedure

The study participants were randomly identified in relation to their hospitalization experience that they had reported to journalists, on television, on social media. The recruitment of the sample, took place through searches from online newspapers, television, public pages of social networks in which the participants gave interviews or wrote their experiences about the hospitalization and the illness. When contact numbers or emails were not immediately available, secondary information (e.g., job, profession, city) emerged from the news was used to deepen web searching and track down people’s phone numbers or email addresses to get in touch with them. A list of 30 potential participants was developed and people were approached by phone or by email to participate in the study. The inclusion criteria were: (1) to have been hospitalized in an ICU for severe conditions due to COVID-19; (2) to have spontaneously told their own experience to journalists, on television, on social media; (3) to freely consent to participating in the study.

### 2.3. Instruments

A semi-structured interview guide was developed by consulting and by adapting instruments from available scientific literature on the topic [8,15,20] and based on the study purpose. Interview questions (Table 1) aimed to understand the emotions felt upon admission, the sources of stress, the feelings experienced, and the current health condition. The interview guide was used flexibly to make conversation natural.

At the end of the interview, the Patient Health Questionnaire (PHQ-4) [30] was administered to the participants to evaluate feelings and emotions about their experience in hospital. The questionnaire included four items adapted to screen for both depression and anxiety feelings experienced by participants during both hospitalization and at the time of the interview. Sample items were: “Nervous feeling, anxiety, nervous wreck” and “Little interest or pleasure in doing things (eating/self-care)”. The items were rated using a four-point Likert scale ranged from 0 (“none”) to 3 (“almost every day”). The values’ sum between 0–2 denote PHQ–4 “normal”, 3–5 “mild”, 6–8 “moderate”, and 9–12 “severe”. Internal reliability of the original scale was good (Cronbach’s Alpha > 0.80). Finally, other useful elements were acquired to describe the hospitalization experience such as the total length of stay in hospital, length of stay in ICU, days of hospitalization in another care area, and having undergone endotracheal intubation. Demographic data such as age, gender, and region were also collected.

### 2.4. Data Collection

A total of 19 out of 30 persons answered the call for the research. Among them, four individuals refused to participate in the study. The final sample included 15 participants. After presentation of the purpose of the research, individual appointments were agreed with the participants and the methods for performing interviews (e.g., via phone or video call) were proposed based on the participants’ preference. The researchers were available to answer to any doubts or clarifications about the project and methods.

Interviews were audio-recorded and had an average duration of 30–40 min. They were always conducted by the same member of the research group. During the interviews, the researcher took written notes to keep track of the key points to be used for data analysis. The interviewer had a Master’s Degree in Nursing Sciences with experience in qualitative interviewing, and they worked within the COVID-19 department for assisting patients with SARS-CoV-2 infection. It was never necessary to interrupt any interview for clinical or other reasons. Participant recruitment and interviews concluded when all data were collected and no new information arrived [31].

### 2.5. Ethics

Participants were informed about the study aims and with prior informed verbal consent, they voluntarily chose to take part in the study and agreed to tape-record their narratives. Anonymous identification code (ID) with sequential number was used for each participant to preserve anonymity. This research complies with the Helsinki and the General Data Protection Regulation (EU) 2016/679 (GDPR). Participants were informed that anonymity would be guaranteed in compliance with the Italian Data Protection Law (Decree No. 196/2003) and their right to withdraw from the study at any time without adverse consequences was clarified.

### 2.6. Data Analysis

All interviews were verbatim transcribed by the interviewer. Slang expressions, variations in voice tone, and silences were reported in the transcripts to emphasize the experiences collected. In a first phase, all members of the research team (the authors of this paper) conducted the transcription analysis separately. Each researcher made a careful reading of each transcript and once they acquired a general sense of the data, each one extracted significant statements and main concepts emerged from the narratives. Any textual notes were written to describe any relevant concern to be discussed with the research group. At the end of this process, each researcher produced a list of some main themes. In a second phase, all the researchers met together and triangulated data by comparing and discussing the results and retracing all the previous steps. After this final discussion, the researchers identified the final main themes.

To strengthen rigor and to minimize potential method bias, the researchers’ reflexivity with respect to their orientations and values was fostered to make them aware that personal and professional background could influence different stages of the research. However, the team was multidisciplinary with adequate expertise to perform the study and manage potential biases. To promote self-reflexivity, field notes with reflections by the interviewer were also discussed and commented with the team during the data analysis, and audits were performed among the team members during the phases of the study.

Quantitative data from PHQ-4 were analyzed in the form of frequencies (n) and percentages, and by analyzing questionnaire scores based on Kroenke et al.’s indications [30].

## 3. Results

### 3.1. Demographic Characteristics

A total of 15 individuals residing in Italy, aged from 38 to 75 years (average age: 56.4 years) represented the study sample. Men (80%) represented most of the study sample. Among them, six aged <50 years, with three women ranged between 63 and 75 years old. Among men, four worked as freelancers, six as employees, and two were retired. Among women, two were housewives and one was retired. Six participants came from Lombardy, a Northern Italy region that recorded the highest percentage of infection cases and hospitalization for severe complications in the first phase of the pandemic. The other nine participants came from regions of Central and Southern Italy, and Islands. Twelve of fifteen (80%) were hospitalized in ICU at the start of the pandemic and three participants were admitted by October 2020. Eleven individuals (73.3%) underwent endotracheal intubation and five of them were also tracheotomized (33.3%) with consequent prolongation of the hospitalization in almost all the cases (Table 2). Clinical management of intubated patients resulted in a longer hospital stay with an average of 71.5 days, if compared with people who were handled with non-invasive ventilation (average hospital stay of 64 days).

### 3.2. Themes Emerged from the Interviews

The analysis of the interviews identified four main themes related to the participant’s experience about ICU hospitalization and the post-discharge period: (i) emotion of fear (split in two sub-themes); (ii) isolation and loneliness; (iii) unawareness about the gravity of the situation as a protective factor; (iv) “Long COVID”: consequences of the disease on health (Table 3).

#### 3.2.1. Theme 1: Emotion of Fear

The emotion mostly reported by participants was the fear related to not being able to overcome the disease and the fear of dying. This emotion was also related to the breathing difficulty that was a complication of the disease, thoughts about not being able to see their loved ones again, and the fear of getting sick again. The theme, given the different facets, was split into two sub-themes: fear for one’s own life and fear about getting sick again (and about the risk of contagion for one’s own family).

##### Sub-Theme 1: Fear for One’s Own Life

For the participants, the fear for one’s own life meant concern for losing their usual life and for not being able to return to their loved ones.

ID7: “the greatest fear was when I got into the ambulance and I thought to my daughter who greeted me from the window saying “Hi dad, I love you”. It was the worst moment, the fear for not coming back home”.

ID9: “the fear for dying, and the fear for not being able to come back my house even for one day… I remember this fear as a daily fear”.

The fear for one’s own life was already real at the time of hospitalization. The participants expressed concern for not being able to overcome the situation with an explicit fear of dying.

ID6: “the first day [of hospitalization] I thought I was going to die. I remembered my whole life and I thought «ok that’s what happened, I am 57 years old and I have already done many things in my life”.

ID15: “fear for failing, I also saw two people die next to me, so the fear of ending up the same was strong”.

ID11: “let’s face it, I saw as a coffin and someone waiting for me”.

The fear of dying emerged even in moments of high emotional tension such as the need for endotracheal intubation was communicated and during the ICU stay, as told by some interviewees:

ID14: “I remember the fear given by the breathing difficulty, fear and panic… and in the end, they [doctors] intubated me (…). After waking up it seemed that the worst was over, but for me, it was not really like this, I always continued to have anxiety and fear to die”.

ID4: “I was very scared, I immediately asked which possibilities I had [with endotracheal intubation]. I was really very afraid, and the greatest fear was to not being able to come back home to my loved ones, afraid to die and not waking up”.

ID15: “the head physician informed me that I needed to be intubated. I would say that I felt terror, I didn’t know what I was getting into (…)”.

##### Sub-Theme 2: Fear about Getting Sick Again and about the Risk of Contagion of Loved Ones

Other forms of fear that emerged by participants’ stories were the fear of having infected their loved ones (during hospitalization) and the risk of their loved ones getting infected (after hospital discharge):

ID4: “one of the biggest fears that never left me was the fear of having transmitted the infection to someone at home”.

ID 11 “(…) Now that the numbers [of infections] are always growing I do not deny that it makes me worry, especially for my grandchildren and the people around me … and this fear I’m only having right now”.

As emerged from the words of two interviewees, together with the fear of contagion of one’s own family, the fear of getting sick again and still being sick was experienced after hospital discharge:

ID8: “(…) to date I am also afraid to infect me again, just afraid of taking it back and reliving everything I have already been through”.

ID13 “(…) When you see such things the fear is still there, since I was discharged I have never had a great social life (…)”.

#### 3.2.2. Theme 2: Isolation and Loneliness

The interviews experienced the feeling of fear in a close connection with that of loneliness due to the impossibility of seeing loved ones, as told by some interviewees:

ID3: (…) well, what could I say … that I was alone, that yes, I suffered loneliness”.

ID7: “I feared loneliness a lot and when a person is alone s/he knows s/he can’t do much, at that moment I raised my hands and said to myself «I know it’s done»”.

ID11: “loneliness seemed an insurmountable thing, thinking about it today I don’t know how I could do it (…). The loneliness was always there, but luckily it was dampened by those wonderful characters [the health care workers] who surrounded me”.

The participants said that the fear of being alone was accentuated by the isolation experienced in the hospital and by the consequent inability to be close to loved ones.

ID6: “There were a few moments where I felt anxious. For example, the lack of contact with people, we were many [hospitalized patients] but perhaps the isolation [from loved ones] made us feel even more alone”.

ID12: “Another thing that I experienced really badly was that we were alone because there were single rooms, it happened that there was not always someone inside, I always asked the nurses what time it was and when it was evening I couldn’t wait to asking for something to sleep, so I could spend some time without thinking I was alone”.

ID13: “I was afraid of being alone, of loneliness… just the fear for not being accompanied, for dying and not having anyone close to, none of the loved one I shared my life with”.

Loneliness was also described as a source of stress and anxiety. Being alone, the need to respect physical distances, and strict mandatory care rules for the prevention of the infection led patients to living isolation in a much more stressful way, regardless of the worry for their health condition, as revealed by three interviewees:

ID4: “(…) the greatest stress was loneliness, being alone when there were no nurses. It was very stressful to me being alone… we had no one close to”.

ID11: “I was alone, I had no company. I had no one, loneliness was the real stress. I wished someone who could stay with me longer, but I understood that it was not possible, I suffered from not having someone to chat with, I missed people, the contact, chatting…”.

ID12: “I remember as if it were yesterday, a day when I burst into tears [with a doctor] (…), but I immediately I said «doctor, it’s not the cure that making me a nervous wreck, because I feel that it [the cure] is going well, but I react like this because I am alone, the fact of not having company makes me very sad”.

#### 3.2.3. Theme 3: Unawareness about the Gravity of the Situation as a Protective Factor

Fear is contrasted with an unawareness of the gravity of the experienced situation. The lack of a concrete perception of the severity of the clinical condition emerged for some circumstances of ICU hospitalization. However, it represented an important aspect that was defined by the interviewees as a positive element.

ID5: “(…) I couldn’t quantify how long I was wide-awake after the coma, perhaps this lack of perception, somehow, saved me”.

ID13: “(…) I don’t remember much of those days in ICU, maybe on the one hand it went better this way… I didn’t realize the gravity of the situation… I would have died of fear (…). On the other hand, I thank a bit the drugs and my freaking out that kept my thoughts away from reality.

ID11: “It seems like as I didn’t have an inkling of what happened to me. I had an inkling that something wasn’t right, but it was as if I had my ears plugged towards what was happening”.

Unawareness of the situation contributed to reducing the perception of the dramatic situation, which countered negative emotions, preventing them from taking over, as emerged from the words of some interviewees.

ID1: “I didn’t know what was going on (…), I didn’t think I was going to die (…), I was never afraid of dying”.

ID7: “(…) there were some days, like those after the coma, where I had no conscience, and maybe that helped me to don’t let me go to bad emotions and feelings”.

ID6: “Everything was so fast and maybe I didn’t become aware of the gravity of what was happening to me. I don’t think I was afraid at that moment [before endotracheal intubation]”.

#### 3.2.4. Theme 4: “Long COVID”: Consequences of the Disease on Health

**Figure 1:**
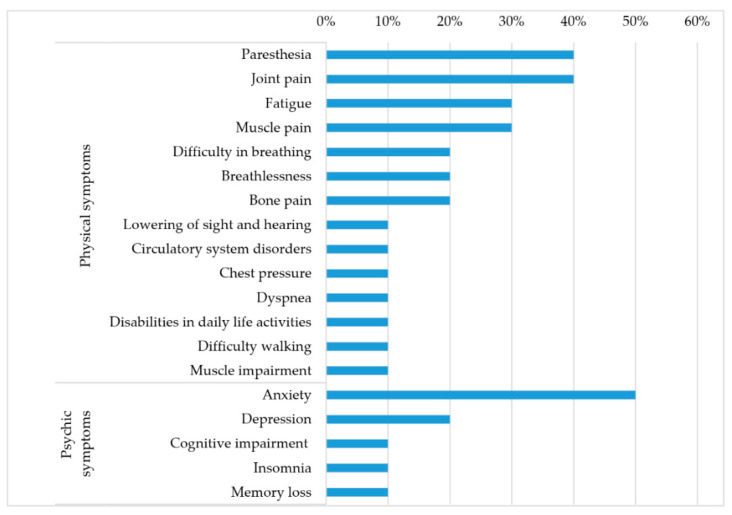
Long COVID symptoms: consequences of the disease on physical and mental health.

The interviewees reported feeling good at the time of the interview, but they listed a number of health problems still present after discharge. Consequences included both a mental dimension and a physical component (Figure 1).

Regarding physical problems, they involve a variety of organs and systems, but the most common consequence included bone, lower limbs, and muscles problems, as evidenced by the words of three interviewees.

ID5: “today I am certainly fine compared to before, but I have problems with my ankles, toes, fingers, and mainly at the bone level (…)”.

ID9: “COVID has left me with pathologies, mainly to my bones and muscles (…)”.

ID11: “(…) I can’t close my hands (…). I can no longer handle the big toe of my right foot; I cannot drive for a year. I have mobility problems, I’ve ruined my hips, when someone sees me walking s/he understands, it’s really hard walking for me”.

In addition, among the problems about the physical level, the participants also reported breathing problems and fatigue:

ID7: “I’m fine now, but COVID has left me with respiratory problems, dyspnea and I feel pressure on my chest (…)”

ID5: “fatigue is always latent and I’m realizing that I no longer have the strength of before (…), I struggle to sing, I’ve never recovered my diaphragmatic breathing”.

ID15: “(…) if I go up the stairs I am a bit out of breath, or maybe when I have to manage bad days, when I feel profound tiredness”.

Again, the interviewees said they have numbness of some parts of the body and difficulty in moving:

ID6: “I have a deficit in the external popliteal sciatic nerve to both feet, a nerve injury and the left thigh still looks like it is under anesthesia”.

ID9: “(…) a circulation problem has arisen in the lower limbs (…). I have a sense of paresis in my feet and legs, my limbs are always cold and I feel electric discharges, as if needles”.

ID14: “(…) neurological problems in the lower limbs, I feel as if they are passing inside the shocks, it seems to feel the electric current”.

In relation to the mental dimension, the interviewees reported problems related to anxiety and memory, as emerged from the words of two interviewees:

ID14: “What I am left is definitely anxiety (…)”.

ID13: “I have problems (…) with my head. Besides anxiety I have many memory lapses, holes, and difficulties in remembering some things that happened a few hours before”.

From the stories that emerged, participants also benefited from psychological or pharmacological support to deal with mental health problems:

ID12: “I’m fine now but mostly on a psychological level I have problems. I always take the sleeping pill and also the psyche pills”.

ID15: “I am not as I was before the hospitalization, but thanks to psychotherapy I am learning to look back as well as forward (…)”.

### 3.3. Emotional and Mental Disorders

Results from the PHQ show that during hospitalization, 10 of 15 participants (66.7%) had mild or moderate depression and anxiety symptoms. After discharge, 13 of 15 participants (86.7%) moved to normal values of patient health. Nobody presented severe symptoms in both hospitalization and after discharge (Table 4).

Table 5 shows participants’ analytical results from PHQ. Specifically, 53.3% (*n* = 8) of participants had anxiety and depression mild values referred to hospitalization and moved to normal values after discharge, one participant (6.7%) moved from moderate to mild values and 40% (*n* = 6) of participants remained unvaried on normal or mild values of anxiety and depression.

## 4. Discussion

This study investigated the experiences and the emotions of COVID-19 survivors who were hospitalized in ICU. Previous studies explored the emotions of patients hospitalized for SARS-CoV-2 infection [8,15,32], but there are still few qualitative studies analyzing the feelings and the experiences of people who survived severe complications of the disease [13,20,33,34], especially in Italy where this kind of study is lacking. The study sample consisted mainly of males and it was representative by gender of the general population admitted to the ICU for COVID-19 in Italian hospitals. In fact, the male gender has a significantly increased risk of ICU transfer [35,36] for managing the most severe complications, and it is associated with the highest percentage of deaths for COVID-19 [36,37]. The results highlighted a complex physical and psychological experience for all the interviewees, both during hospitalization and after discharge at the time of the interview. The participants found themselves in an unexpected, severe, and life-threatening condition by a virus of which, at the time of the study, still little or nothing was known. Negative feelings, fear, and loneliness were often experienced. Fear was described as worry for one’s own life, a fear of dying, and not being able to overcome the unknown disease. Moreover, in the interviewees’ stories the fear of not coming back to loved ones and having them infected; fear of getting sick again and reliving the dramatic experience of hospitalization also emerged. This emotion was probably because in Italy the vaccine was not yet available for citizens, and fear of new contagion was high. Fear in all its facets is a feeling emerging in recent studies. Specifically, the fear of dying [8,15,21], being intubated [20], being infected again [34], and infecting family members [33,38]. Furthermore, respondents reported that they had experienced anxiety associated with fear of dying and fear for the deterioration of their abilities due to the disease. Indeed, providing the patient and their families with clear and appropriate information about their health by healthcare professionals can help patients to cope with their uncertainties and fears during hospitalization [39]. The anxiety was also linked to the loneliness suffered in the hospital due to the impossibility of meeting loved ones and the difficulty of communicating with them. In fact, interviewees were forced into isolation due to restrictions to limit the spread of the infection. Loneliness was also amplified by the fact that patients were alone in the room and they could not talk to anyone. Additionally, contact with professionals was minimal, thus feeling even more alone. Participants’ feelings and emotions changed from the period of hospitalization to the post discharge period. During ICU hospitalization, most of the participants experienced feelings of sadness, depression, or loss of hope; after discharge, at the time of the interview, some participants still had these feelings. Almost all interviewees experienced anxiety and it persisted, although to a lesser extent, during post discharge for more than half of them. Similarly, a study conducted in China indicated that hospitalized patients recovered from COVID-19 showed significant improvement in symptoms of depression and anxiety one month after discharge [11]. The interviewees said that during their hospitalized in the ICU, fear was fueled by various physical problems, in particular by the difficulty in breathing normally. Studies on patients’ experiences of dyspnea, prior to the COVID-19 pandemic, indicated that severe breathing difficulties had a profound impact on patients, thus resulting in feelings of distress and fear of dying during hospitalization in the ICU [40], especially during and after the acute episode of dyspnea [41]. In such a situation, it is important that healthcare professionals provide support to patients. Useful nursing actions such as comfort, companionship, fostering self-concentration, and supplying information were identified from literature [41]. Furthermore, the interviewees referred fear to specific moments such as hospitalization, transfer to ICU and when healthcare professionals needed to proceed with endotracheal intubation. To increase confidence and to minimize fear of the illness, communication with patients should be contextual to the different phases of the hospitalization. Diagnosis, clinical care, and nursing care activities should always be consistent with each other and patients should always be heard when they reference any problem or need [39]. Most of the survivors reported that during ICU admission, fear was associated with loneliness and it was described as a fear of being alone. Loneliness emerged as a negative feeling, and it was referred to be a source of stress during the hospital stay; this is in line with the study by Sun et al. [15] Likely, the loneliness was further amplified by forced isolation in the hospital and the consequent distance from loved ones. The inability to receive visits to the hospital had a negative impact on the interviewees, which sometimes was relieved by the possibility of contacting family by telephone or video call. As isolation to contain the risk of infection can have negative consequences for segregated patients [42], fostering the use of communication instruments such as smartphones and video chats would decrease the sense of isolation [16,39]. Moreover, more than one-third of the survivors referred that establishing friendly relationships with other inpatients and healthcare staff was very important for them. These aspects are also emphasized in other studies according to which family and social support are recognized to be important resources during the hospital stay [15,19], as well as expanding social contacts with other patients to comfort each other during isolation from external people [13]. The interviewees said that loneliness was lessened by the interaction (albeit brief) with healthcare professionals, in particular with nurses, and that their presence was a source of relief from loneliness. In effect, as found in previous studies, the empathic attitude of nurses and other staff members can improve patients’ mood [38] and decrease their feelings of fear and loneliness [21]. In this way, healthcare professionals should reflect on care time to dedicate to patients as an integrative part of the caring process. Furthermore, the psychologist figure would be important to integrate in the care team to promote physical and mental recovery for COVID-19 survivors [8]. Therapeutic sessions should begin during hospitalization, when patients are clinically stable, and continue after discharge [10] to reduce the risk for patients to develop psychological morbidity [20].

An interesting aspect that emerged from the interviewees’ stories was their unawareness of the gravity of the experienced situation, which is opposed to the negative feelings experienced by survivors. In effect, many interviewees reported the lack of a concrete perception of criticality of their clinical condition during hospitalization, an aspect defined by themselves as a positive element. Unawareness would therefore represent a protective factor that attenuates the perception of the drama of the lived experience, thus preventing negative emotions from taking over.

However, some interviewees who did not remember anything about the lived experience said that they would have liked to remember to allow them a better awareness of what happened. This aspect is in line with the results of Mongodi et al. [25] in which survivors reported having nightmares and an initial belief that they were in a fake hospital, such that in some cases they wanted to see the ICU to help rebuild their memory.

The most important contribution of this study is the relevance about the long-COVID problem. This aspect emerged from the interviews, and it is unique to COVID-19 for all hospitalized patients. It refers to post-discharge complications and the long-term effects of the disease. The interviewees reported negative consequences affecting different body districts and the psychic sphere. Although they reported an improvement in their health condition if compared to the hospitalization period, the participants reported that they still had fatigue, dyspnea, wheezing, depression, insomnia, and memory impairment in some cases. The World Health Organization considered these symptoms as common after the illness, and they usually had an impact on quality of life [6]. Furthermore, such symptoms were also found in patients from other studies on the subject [43,44], thus strengthening our results. On a physical level, in addition to fatigue after short efforts, most of the survivors reported various problems and deficits of both the musculoskeletal and the nervous systems. In particular, prolonged bed rest and the use of pharmacological sedation may have resulted in outcomes at motor level. In fact, several participants reported difficulty in walking, partial pain and paresthesia in the lower limbs even for more than four months after discharge. These findings are supported in literature and confirm that the long-term effects of the virus persist after discharge [45]. Furthermore, the summary of the complications reported by the interviewees showed a substantial coincidence with the symptoms of Post Intensive Care Syndrome (PICS). Cognitive impairment, disabilities in life activities, and daily disability occurred after critical illness and for up to 12 months after discharge from the ICU [23]. Although there is still a need for studies exploring clinical characterization of patients with long-COVID to provide appropriate treatments, the literature shows the existence of risk factors (e.g., age, comorbidity, hypertension, obesity, etc.) for not returning to “usual health” [46]. As long-COVID impacts patients’ quality of life [47], it is important to recognize patients at risk to ensure effective take charge of the patient from discharge and to provide continuity of care in the community.

Regarding quantitative results from the PHQ, the majority of participants had mild or moderate values of anxiety and depression referred to the hospitalization period and then changed to normal values after discharge. Nobody presented severe mental health symptoms in both hospitalization and after discharge. These results suggest that feelings of anxiety and depression seems to be more related to the experience of uncertainty and isolation due to hospitalization, and once patients return to their personal life routine, these feelings lessen despite long COVID effects. However, although 13 out of 15 participants refer a reduction in mental health-related disorders after discharge, recent findings emphasize that long-COVID symptoms negatively impact quality of life [47], thus highlighting the importance for a holistic take in charge prior to patient discharge for his/her care continuity in the community. It is important to note that the results of the present study refer to the first wave of the pandemic. This suggests that the psychological symptoms present during participants’ hospitalization were referred to a period in which the infection was perceived as severe and from which people died. This perception inevitably increased people’s insecurity and probably exacerbated the psychological symptoms that seemed to decrease after discharge. However, a lot is already known now about the disease, while there are still few studies on the mental health of people recovered from COVID-19, especially those with long COVID symptoms for whom there is an increased likelihood of developing psychiatric pathologies and mood disorders. [48].

This research had limitations that should be considered for future studies. Due to random sampling, the female gender was poorly represented. However, this aspect was in line with what emerged from the general Italian population hospitalized in the ICU for COVID-19. Indeed, the male gender had a higher risk of transfer to the ICU [36] for the management of the most severe complications. Also, the sample included people who voluntarily made their experience public; in this sense, the recruited participants may be more motivated and have a higher response capacity to critical events than other people with a similar experience. Furthermore, in order to comply with the pandemic emergency regulatory restrictions and to meet the participants’ needs, data were collected via phone interview or video call. Thus, in some cases, it was not possible to integrate the verbal message with the non-verbal language of the participants. Some barriers to communication (e.g., tracheostomy and deafness of the interviewees) may have made it difficult to interact with the interviewer, and it may have affected the quality of the data collected. Finally, like all qualitative studies, generalizability of the results was largely inapplicable and the findings of this study should be transferred with caution, as well as the quantitative data due to the poor sample size.

Based on the study results, future research may focus on long-COVID by investigating people’s real care needs and how professionals can support them to better cope in the post-COVID phase, thus improving quality of life. In addition, it may be interesting for future studies to compare the perception of survivors’ PHQ before getting COVID with perceived PHQ during and after hospitalization.

## 5. Conclusions

The results indicated that most of the study participants admitted to the ICU for the SARS-CoV-2 virus infection mainly experienced feelings such as fear, loneliness, and anxiety during hospitalization, and they had physical and psychological consequences after discharge from the hospital (long-COVID). Based upon the recent Italian law 77/2020 that defines a nurse role as important to manage COVID-19 patients in the community, it is crucial to identify patients at risk for long-COVID and guarantee a holistic take in charge starting before the discharge and continuing care after discharge in the community where they live.

## Figures and Tables

**Table 1:** Semi-structured interview guide.

Questions
1. How did you feel when you realized (or were told) that you were hospitalized in ICU?
2. What was your main worry/stressor during ICU hospitalization?
3. How is your health now (any consequences)?
4. What was your feeling about becoming seriously ill during this pandemic?

**Table 2:** Participants’ characteristics and length of stay (LoS).

Participant ID	Gender	Age	Respiratory Support in ICU	ICU LoS (Days)	Total LoS (Days)
1	M	41	TI	18	21
2	M	57	TI + tracheostomy	98	159
3	M	61	TI	29	65
4	M	38	TI	10	45
5	M	48	TI + tracheostomy	45	90
6	M	49	TI	34	90
7	M	57	TI + tracheostomy	19	41
8	F	63	NIV/mask ventilation	13	27
9	F	75	NIV/mask ventilation	42	90
10	F	65	NIV/mask ventilation	45	84
11	M	70	TI + tracheostomy	54	120
12	M	66	NIV/mask ventilation	40	55
13	M	65	TI	22	40
14	M	43	TI + tracheostomy	48	108
15	M	48	TI	21	38

Note. TI = Tracheal intubation; NIV = Non-invasive ventilation.

**Table 3:** Main themes emerged from the interviews.

N.	Main Themes	Interviewees from Who the Themes Were Emerged
i	Emotion of fear	ID4, ID6, ID7, ID8, ID9, ID11, ID13, ID14, ID15
	- Fear for one’s own life	ID4, ID6, ID7, ID9, ID11, ID14, ID15,
	- Fear about getting sick again and about the risk of contagion of loved ones	ID4, ID8, ID11, ID13
ii	Isolation and loneliness	ID2, ID3, ID4, ID6, ID7, ID10, ID11, ID12, ID13
iii	Unawareness about the gravity of the situation as a protective factor	ID1, ID5, ID6, ID7, ID11, ID13
iv	“Long COVID”: consequences of the disease on health	ID5, ID6, ID7, ID8, ID9, ID11, ID12, ID13, ID14, ID15

**Table 4:** Participants’ emotional and mental feelings from PHQ.

**PHQ Referred to the Hospitalization Time**		
**Score/Value**	**Participant (*n*)**	**%**
0–2 (normal)	5	33.3%
3–5 (mild)	9	60%
6–8 (moderate)	1	6.7%
9–12 (severe)	0	0
Total	15	100%
**PHQ Referred to the Time of the Interview**		
**Score/Value**	**Participant (*n*)**	**%**
0–2 (normal)	13	86.7%
3–5 (mild)	2	13.3%
6–8 (moderate)	0	0
9–12 (severe)	0	0
Total	15	100%

Note. PHQ = patient health questionnaire.

**Table 5:** Individuals’ score on mental health symptoms from PHQ.

ID Participant	PHQ Value during the Hospitalization (Sum)		PHQ Value at the Time of Interview (Sum)	
1	5	(mild)	0	(normal)
2	2	(normal)	1	(normal)
3	0	(normal)	0	(normal)
4	3	(mild)	2	(normal)
5	1	(normal)	0	(normal)
6	1	(normal)	0	(normal)
7	4	(mild)	1	(normal)
8	5	(mild)	3	(mild)
9	1	(normal)	1	(normal)
10	4	(mild)	0	(normal)
11	5	(mild)	1	(normal)
12	3	(mild)	1	(normal)
13	4	(mild)	1	(normal)
14	7	(moderate)	3	(mild)
15	5	(mild)	2	(normal)

## Data Availability

The data presented in this study are available on request from the corresponding author. The data are not publicly available due to privacy reason.
